# Papillary Muscles of the Left Ventricle: Integrating Electrical and Mechanical Dynamics

**DOI:** 10.3390/jcdd12010014

**Published:** 2024-12-31

**Authors:** Csilla Andrea Eötvös, Teodora Avram, Roxana Daiana Lazar, Iulia Georgiana Zehan, Madalina Patricia Moldovan, Patricia Schiop-Tentea, Giorgia Coseriu, Adriana Sarb, Gabriel Gusetu, Elena Buzdugan, Roxana Chiorescu, Diana Mocan-Hognogi, Sorin Pop, E. Kevin Heist, Dan Blendea

**Affiliations:** 1 Niculae Stancioiu Heart Institute, University of Medicine and Pharmacy “Iuliu Hatieganu”, 400001 Cluj-Napoca, Romania; csilla.andrea18@gmail.com (C.A.E.); teodora.avram97@yahoo.com (T.A.);; 2 Rehabilitation Hospital, University of Medicine and Pharmacy “Iuliu Hatieganu”, 400066 Cluj-Napoca, Romania; 3 Municipal Hospital, University of Medicine and Pharmacy “Iuliu Hatieganu”, 400139 Cluj-Napoca, Romania; 4 Emergency Clinical County Hospital, University of Medicine and Pharmacy “Iuliu Hatieganu”, 400347 Cluj-Napoca, Romania; 5 Demoulas Center for Cardiac Arrhythmias, Massachusetts General Hospital, Harvard Medical School, Boston, MA 02215, USA; kheist@mgh.harvard.edu

**Keywords:** papillary muscles, arrhythmia, mitral valve, dyssynchrony, mitral regurgitation

## Abstract

Background: Papillary muscles are structures integrated into the mitral valve apparatus, having both electrical and mechanical roles. The importance of the papillary muscles (PM) is mainly related to cardiac arrhythmias and mitral regurgitation. The aim of this review is to offer an overview of the anatomy and physiology of the papillary muscles, along with their involvement in cardiovascular pathologies, including arrhythmia development in various conditions and their contribution to secondary mitral regurgitation. Methods: A literature search was performed on PubMed using the following relevant keywords: papillary muscles, mitral valve, arrhythmia, anatomy, and physiology. Results: During the cardiac cycle, papillary muscles have continuous dimensional and pressure changes. On one hand, their synchrony or dyssynchrony impacts the process of mitral valve opening and closure, and on the other hand, the pressure changes can trigger electrical instability. There is increased awareness of papillary muscles as an arrhythmic source. Arrhythmias arising from PM were found in patients with or without structural heart disease, via Purkinje fibres, due to increased automaticity or triggered activity. Conclusions: Despite the interest in mitral valve physiology, there are still many unknowns in relation to the papillary muscles, especially with regard to their role in arrhythmogenesis and the pathogenesis of mitral regurgitation.

## 1. Introduction

One of the first descriptions of the papillary muscles (PM) of the left ventricle (LV) dates back to the Renaissance era, when Leonardo da Vinci, while dissecting the heart of an ox, noticed the existence of the PM attached to the ventricular walls. He described two potential roles for these structures: preventing the LV cavity from collapsing during systole, allowing the blood to pass from the right ventricle to the LV via the interventricular septum, and anchoring the ventricular walls in place and preventing excessive dilatation of the LV cavity during diastole [1].

## 2. Anatomy

The LV has two PMs, anterolateral and posteromedial, located on the anterolateral and posteromedial walls, respectively, usually at the midventricular level. These anatomical structures are components of the subvalvular apparatus of the mitral valve (MV), which facilitates unidirectional blood flow inside the heart [2].

**Figure 1:**
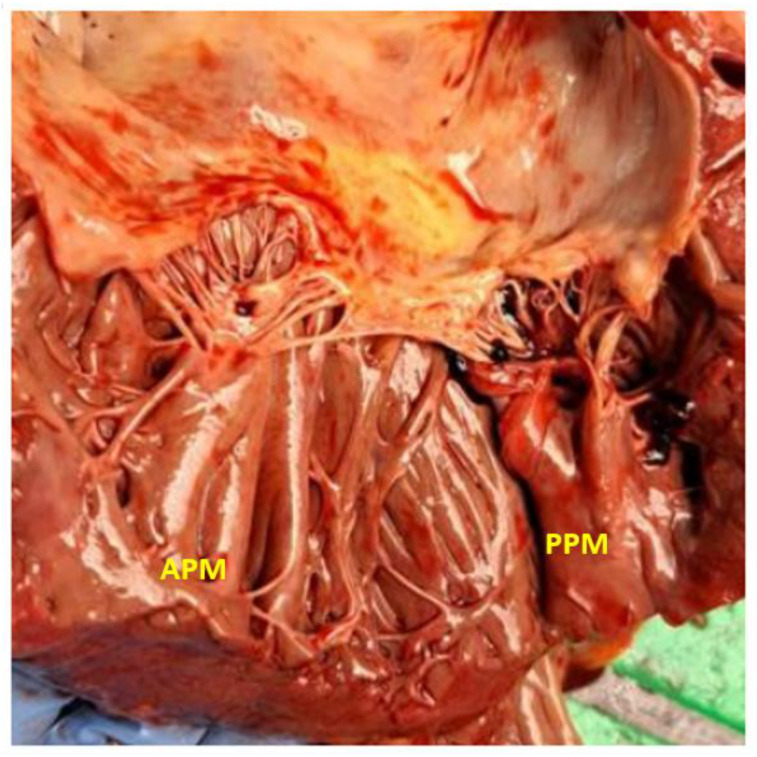
Anterolateral (APM) and posteromedial (PPM) papillary muscles in a necropsy specimen (courtesy of Dr. Doinita Crisan).

The morphology of the PM is highly variable in shape, number, and attachments [3]. Most anatomical reports describe the PM as being composed of a trunk with multiple heads averaging about six per PM [4]. The anterolateral PM is usually single, as opposed to the posteromedial PM, which can have two or more trunks (up to five) [5]. The PM can be either ‘tethered’ or ‘finger-like’. The tethered PMs are attached to the LV free wall via several trabecular ridges (see the anterior papillary muscle (APM) in Figure 1), while the finger-like papillary muscles are usually attached to the LV free wall over a broad base (see the posterior papillary muscle (PPM) in Figure 1) [6,7]. In addition, there are descriptions of various interconnections between the heads of the same PM and even between the two groups across the ventricular cavity (false tendons, muscular bands) [5,8].

False tendons are fibrous or muscle structures, variable in length and thickness, found in the LV cavity, generally located between the free wall of the LV or a PM and the interventricular septum, without connection to the mitral valve [9].

The variability in morphology and interconnections could be explained to a certain extent by taking into consideration the embryological development of the heart [10]. Papillary muscle development begins at week five in utero, with the muscular trabecular ridge. Between 8 and 10 weeks of development, due to delamination of the ventricular myocardium and detachment from the ventricular ridge, the PMs become freely movable structures [10]. It has been mentioned in several studies that each PM has its own unique morphology, being likened to a fingerprint [5,11,12].

## 3. Ultrastructure of the Papillary Muscles

From a histological point of view, the PM has two layers: myocardium and endocardium, the latter being composed of endothelial and subendothelial layers covering the PM. Myocardial fibres have a complex architecture, with multiple myocardial bundles that intersect, which can result in an anisotropic depolarization of the PM [6]. The left bundle branch of the His, which runs in the interventricular septum and generates the anterior and posterior fascicles, ultimately subdivides into a fascicular network which allows synchronous activation of the LV, including that of the PMs [8].

False tendons are chord-like structures, which lack the insertion point to the mitral valve, but they frequently have one or sometimes two attachment points to the PMs of the LV. Histologically, they can be fibromuscular or muscular bands, but often they contain conduction tissue similar to those observed in the His bundle, raising the question whether these structures are a part of the conduction system [13]. The presence of these PM connections, whether between the PM and the ventricular wall or between the two PMs, may be associated with atypical exit sites for PM arrhythmias. These sites, which may be located away from the typical base of the PM, could explain the variability in ventricular activation patterns, as reflected in the QRS axis and/or the precordial transition zone [6,14].

Purkinje fibres are located predominantly at the level of the subendocardial layer with the network being more dense at the level of insertion of the PM to the ventricular wall, determining the activation of the PM from base to apex [15]. Additionally, the intracavitary position of the PMs themselves relative to the septum and the left ventricular free wall was found to be correlated with the QRS duration in healthy subjects [16].

The electrical activation of the LV PMs occurs at the initial phases of the electrical activation of the ventricles. According to Armour et al., who studied electrical and mechanical PM activation in dogs, there is a delay of approximately 45 ms between the electrical activation of the anterior PM and the mechanical contraction of the surrounding epicardium. Myocardial contraction in both PMs begins approximately 20 ms later, occurring during an intraventricular pressure rise and reaching maximum tension after the beginning of ventricular systole. A rapid decrease in intraventricular pressure is followed by the relaxation of the PM [17].

## 4. Physiology

Although they are complex structures, PMs are not independent entities but are part of the mitral apparatus, which also includes chordae tendineae, the mitral valve, the mitral annulus, and the adjacent LV and left atrial (LA) walls.

During the cardiac cycle, PMs have movements that are synchronous with rest of the mitral apparatus [5]. This applies to circumferential as well as longitudinal contraction and relaxation. Sanfilippo et al. [18] have shown using echocardiography that in normal subjects, during systole, both PMs and the mitral annulus are displaced toward the LV apex, and the distance between the PM tips and the mitral annulus remains relatively constant. In mitral valve prolapse, the mitral leaflets are displaced in systole towards the left atrium and, as a consequence, they apply increased traction to the PM via the chordae tendineae, which causes systolic displacement of the PMs towards the annulus (instead of maintaining a constant distance as is the case with normal subjects). A possible consequence of this increased traction force applied to the PM is proarrhythmia [18,19].

**Figure 2:**
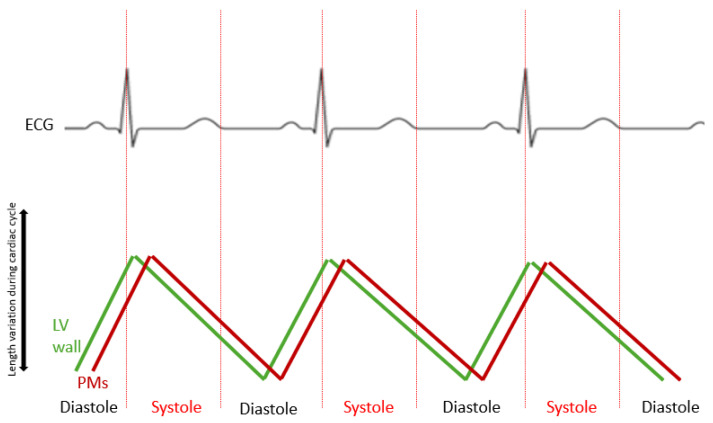
Papillary muscle (PM) length variation during cardiac cycle in contrast with left ventricular wall (LV wall). The electrical activation of the left ventricular papillary muscles occurs during the early stages of ventricular activation, while their mechanical contraction happens about 20 ms after the contraction of the surrounding epicardium (Armour et al. [17]).

In their experimental study in dogs, Marzilli et al. [20] measured the dimensions of the LV PMs during the cardiac cycle in order to determine their dimensional change and their impact on the mitral valve biomechanics. Maximal shortening and elongation of the free wall segment of the LV preceded the corresponding dimensional changes of the PM. The PM shortening began in the ejection phase and continued throughout systole and into diastole, towards the end of isovolumic relaxation. The PM lengthened during late diastole, isovolumic contraction and a short portion of the ejection phase (Figure 2) [21]. Shortening of the PM at the time of isovolumic relaxation, by increasing the tension in the chordae, facilitates the process of mitral valve opening (Figure 2). These dimensional changes together with the integrity of the mitral valve apparatus will permit a proper function of the mitral valve in open and closure, preventing its prolapse and giving an active character to this process [20].

Despite the numerous articles published, the precise role of the PM in the opening and closure of the mitral valve remains to be fully defined. Further studies on this topic may help to clarify the degree of PM involvement in primary and secondary mitral regurgitation.

## 5. Arrhythmia Originating from the Papillary Muscles

The last decade has brought about increased awareness about PMs as an arrhythmic source in both structurally normal and abnormal hearts [3,15,19,22].

Papillary muscle arrhythmias can originate in the regular myocytes or in the Purkinje network, and are thought to be caused by delayed afterdepolarisations (triggered activity) or abnormal automaticity [8]. The site of origin of ventricular arrhythmias was often found to be at the Purkinje–fibre–muscular interface [23].

The mechanical stress experienced by the PMs throughout the cardiac cycle may increase the risk of developing arrhythmias, particularly in patients with mitral valve prolapse, as well as in those with other types of structural heart disease [19]. The mechanical stretch-mediated mechanism, however, is less likely to be involved in ventricular arrhythmias occurring in patients with normal LV size and function and without structural abnormalities. In these cases, arrhythmogenesis was attributed to interactions at the Purkinje–myocardial junction [23].

Decreased Purkinje cell–ventricular myocardium coupling at the PM might be responsible for arrhythmogenesis via increased automaticity or triggered activity.

In addition, abrupt changes in fibre orientation, which occur at the Purkinje network–myocardium interface at the base of the PM, can create conduction delays and micro-reentry and thus also play a role in arrhythmogenesis [24]. PMs in animal studies were found to be located at the centre of reentrant wavefronts and play a role in the pathogenesis of ventricular tachycardia or ventricular fibrillation [25]. Ventricular tachycardia can be generated at the PM level via a macro-reentry scar-related mechanism as well, in which case it is typically inducible with programmed stimulation.

Ventricular ectopy originating in the PMs is typically benign, but there have been cases when ventricular fibrillation (VF) was triggered by short-coupled premature ventricular complexes in patients with structurally normal hearts as well as in patients with coronary artery disease or mitral valve prolapse [22,25,26,27,28].

In mitral valve prolapse, the PMs’ movement towards the mitral annulus as a consequence of the prolapse causes an abnormal tension in the PM’s body, which can turn into electrical instability [18]. The electrophysiologic changes can lead to ventricular arrhythmias even before areas of fibrosis are present on contrast-enhanced cardiac magnetic resonance imaging (MRI) [15]. These complex ventricular arrhythmias may be related to stretch-mediated activity rather than a fixed myocardial scar [29]. A predominance of PM site origin regarding ventricular arrhythmias was observed among women during electrophysiologic studies [30].

As mentioned, myocardial fibrosis, identified by cardiac MRI, can cause premature ventricular contractions. This fibrosis results from mitral valve prolapse stretching the myocardium and PMs, seen as late gadolinium enhancement [30,31,32]. Complex ventricular arrhythmias and a higher frequency of premature ventricular contractions and non-sustained ventricular tachycardia were observed in patients with sudden cardiac death who had considerably more fibrosis than those without [29,33,34]. In their review, Malagoli et al. attempted to develop an imaging-based algorithm to assist clinicians in identifying patients with MVP who are at a higher risk of developing malignant ventricular arrhythmias [32]. By applying the speckle-tracking technique, they recommended that patients with abnormal left ventricular longitudinal strain on echocardiography should be further assessed using CMR to identify fibrous regions [32].

An experimental study found that QRS morphology was different from the baseline QRS after the traction of the PM in the late diastolic phase. This alteration in QRS morphology was related to early activation in the nearby area of the tractioned zone, as a consequence of mechanical stress of the PM or distortion of local Purkinje fibres, generating transient afterpotentials [19].

Several studies have revealed that bileaflet mitral valve prolapse, PM fibrosis, and mitral annulus disjunction increase the risk of cardiac arrest through ventricular arrythmias [31,32,33,35,36]. Mitral annulus disjunction represents systolic separation between mitral annulus and the basal posterior wall of the LV, and a self-sufficient arrhythmogenic entity [37]. It is postulated that mitral annulus disjunction leads to excessive mobility of the MV apparatus, which, in turn, causes traction on the PMs and the posterobasal LV myocardium, serving as the trigger for ventricular arrhythmias. This increased traction causes repetitive mechanical injuries to the myocardium, activating apoptosis pathways and inducing PM and LV fibrosis (the arrhythmic substrate) [33]. Both localised and generalised ventricular remodelling, as well as diffuse fibrosis, were identified as being linked to patients with MVP [38,39]. The combination of a substrate and a trigger provokes early after-depolarisations, which can lead to premature ventricular complexes, ventricular arrhythmias, and sudden cardiac death [40].

Dejgaard et al. [37] discovered that curling of the base of the lateral ventricular wall was associated with malignant ventricular arrhythmias in 12% of the cases and in 22% of the patients, mitral valve prolapse was not present concomitantly, palpitations being by far the most persistent clinical feature. They also observed that mitral annulus disjunction was present at cardiac MRI up to 2/3 of mitral annulus circumference, with a variable longitudinal dimension between 1 mm and 15 mm, bounded by normal, non-disjunctive tissue [37]. Despite the association with sudden cardiac death, mitral annulus disjunction remains an entity with clinical relevance not yet fully elucidated, emphasising the need to develop sudden cardiac death risk scores to assess the individual risk, allowing for the subsequent management of each of these patients.

In another study focused on MVP and mitral annulus disjunction, Cerere et al. [41] assessed the presence of late gadolinium enhancement on cardiac MRI and aimed to quantify the degree of myocardial fibrosis in this patient group using a five-standard deviation gray-scale threshold on semi-quantitative late gadolinium enhancement MRI. This method yielded a percentage of myocardial fibrosis, proving to be a reproducible technique that closely aligned with visual operator analysis. It allowed for more accurate identification of patients with varying degrees of late gadolinium enhancement, potentially associated with ventricular arrhythmias in those with MVP. Increased fibrosis in the PMs and annular region was linked to the occurrence of ventricular arrhythmias [41].

Another morphofunctional aspect of mitral valve prolapse which is correlated with increased risk of developing ventricular arrhythmias encompasses the high velocity of the mitral annulus (Pickelhaube sign) [29]. Together with mitral annulus disjunction, they have a role in the paradoxical increase in mitral annulus diameter during systole, being present in patients with arrhythmogenic mitral valve prolapse [31]. Transposed into practical terms, the high velocity of the posterolateral portion of the mitral annulus is an indicator of increased tension on the PM, providing an echocardiographic parameter useful for the assessment of arrhythmogenesis induced by PM and myocardium stretch, with triggered activity serving as an electric substrate [29,31,37,42].

Although most patients with mitral valve prolapse have a normal ECG, there are cases where some ECG features indicate a high risk of ventricular arrhythmias: QT prolongation, T wave abnormalities (biphasic, inverted), increased QT dispersion [29]. T wave abnormalities in inferior leads have been observed to be present on the ECG of patients with MVP and cardiac arrest, probably serving as a hallmark for the increased mechanical stress exerted on the PM and underlying myocardium by the prolapsed valve [29,33,34,43]. Increased QT dispersion is associated with heterogeneity in repolarisation [44] resulting from local variations in action potential and is strongly associated with arrhythmic events [45].

**Figure 3:**
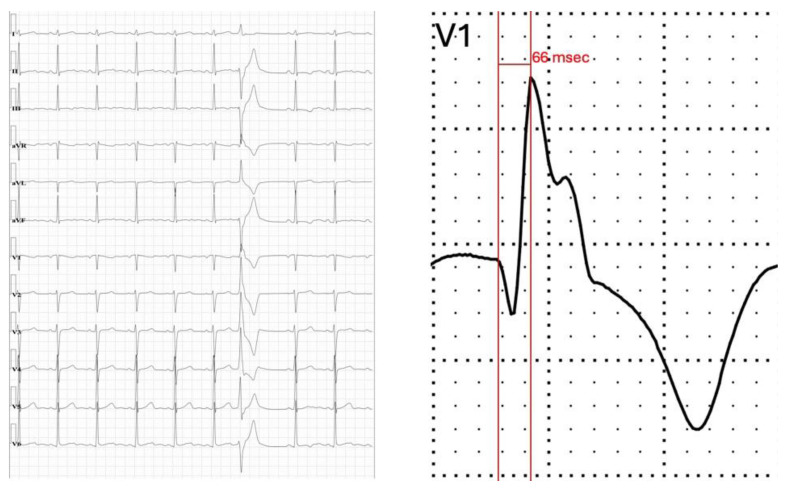
Characteristic electrocardiographic pattern for ectopy originating from the posteromedial papillary muscle. The superior axis and qR pattern, with slurred R wave and short intrinsic deflection (66 ms), in lead V1 is notable. In addition, there is a late transition in the precordial leads. Electrocardiogram from a patient with mitral valve prolapse.

Ventricular arrhythmias (VAs) originating at the level of the PMs typically have a right bundle branch block (RBBB) pattern on the ECG in lead V1 with a short intrinsic deflection and a variable precordial transition [46]. This early intrinsic deflection may be related to the proximity of the PMs to the conduction system [46]. In the case of VAs from the posteromedial PM, they typically have a qR pattern in lead V1 with a short intrinsic deflection (Figure 3), while VAs from the anterolateral PM have a rightward/inferior axis and, in some patients, discordance between the inferior leads, with lead II being negative and lead III positive [46].

Currently non-invasive mapping of the arrhythmia source is performed using a 12-lead ECG. However, in order to improve location accuracy, future strategies may benefit from computational methods for noninvasive preprocedural mapping [47]. The electrocardiographic differential diagnosis of the PM arrhythmias includes arrhythmias originating in the surrounding structures, which include the anterior and posterior fascicles, outflow tract, and mitral annulus. Mitral annulus VAs have inferior lead discordance and positive concordance in the precordial leads [46,48].

Given the specific Purkinje network and myocardial fibres disposition at the level of the PMs, there is anisotropic and preferential conduction, which is responsible for different QRS morphologies during pacing or during VAs originating from the PMs. This is an important feature in differentiating PM arrhythmias from fascicular arrhythmias, the latter exhibiting the same morphology due to the involvement of the normal conduction system and reentrant mechanism [49]. Another important distinguishing characteristic of these two origins of ectopy is the QRS duration: if it is less than 130 ms, the origin is fascicular with a sensitivity and specificity of 100% [7].

Briceno et al. [46] reported that the intrinsicoid deflection in V1 in PM premature ventricular contractions occurred significantly earlier in comparison with other sites of origin (63 ± 13 ms vs. 79 ± 24 ms); an intrinsicoid deflection of less than 74 ms had a sensitivity of 79% and a specificity of 87% for PM ectopy. This observation reinforces what other studies [15,50] have already concluded: the existence of a dense subendocardial layer of Purkinje fibres, which explains the relatively fast intrinsicoid deflection due to the proximity between the conduction system and the PM.

Catheter ablation of PM arrhythmias is a challenging procedure for several reasons. First, the heterogeneous morphology of the PMs and their continuous motion inside the LV cavity make achieving catheter stability during ablation very difficult. In addition, the location of the arrhythmic foci deep within the body of the PM and the varied exit points are a challenge, requiring accurate pace mapping as well as activation mapping [6,51,52]. Thirdly, there is a potential for damaging the PM during the ablation procedure with consequent mitral regurgitation and subsequent recurrence of arrhythmia [6]. A study published in 2023 investigated the factors influencing the heterogeneous morphology of PVCs and ventricular arrhythmias originating from PMs [52]. The researchers categorised the sites of arrhythmia origin into five distinct groups and concluded that the pleomorphism was linked to the varying origins of premature ventricular contractions and the preferential conduction through branching sites of the PMs [52].

Ablation strategies were adapted for the specifics of arrhythmias originating in the PMs including the use of irrigation catheters, optimising the depth of the lesion and tissue-catheter contact (radiofrequency or cryoablation), but this procedure remains a real challenge for the interventional electrophysiologist [51,52].

## 6. Mitral Valve and Papillary Muscles’ Synchrony

**Figure 4:**
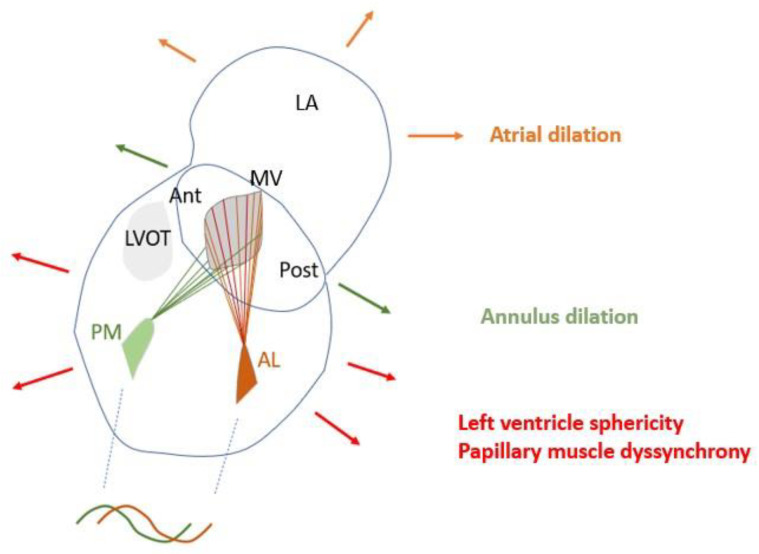
Elements involved in genesis of mitral regurgitation: atrial dilation, annulus dilation, papillary muscle dyssynchrony, left ventricular dilation. (LA: left atrium, MV: mitral valve, Ant: anterior mitral leaflet, Post: posterior mitral leaflet, LVOT: left ventricular outflow tract, PM: posterior papillary muscle, AL: anterior papillary muscle).

The mitral valve apparatus is one of the most complex structures in human body, having two parts: the mitral valve annulus with the two leaflets and the subvalvular apparatus composed of the PM which are attached to the LV free wall, and chordae tendineae, which make the connection to the leaflets. Located between the left atrium and ventricle, these anatomical structures are strongly interconnected; therefore, an alteration affecting any level will lead to mitral regurgitation (atrial, primary or ventricular mitral insufficiency) (Figure 4).

The PMs play a role in the opening and closing of the MV. As already mentioned, PM depolarisation begins early in the cardiac cycle, and their shortening occurs at the time of isovolumic contraction [17]. Probably, these dimensional changes may assist a constant annulus–PM tip distance to prevent mitral valve prolapse [18].

In healthy subjects, LV contraction was accompanied by mitral annulus and PM movement toward the apex during systole, maintaining a constant distance between PM and mitral annulus, preventing atrial displacement of the MV [18]. During diastole, more precisely during isovolumic relaxation, the PMs get shorter and open the MV and are probably accompanied by the motion of the LV wall outwards [20,53]. According to Marzilli et al. [20], who studied the PM in dogs, the timing of contraction and relaxation is slightly different in the PM and the LV free wall. Papillary muscle tension does not appear to parallel the changes in dimensions of the PM. The highest tension in the PM and chordae occurred early during the ejection phase when the PM was maximally elongated. Conversely, low tension occurs in early diastole when the length of the PM is at its minimum [20]. Optimal function of the MV requires the integrity of all structures involved in this active process and is a result of the interaction between the leaflets and surrounding myocardium [54].

The left bundle branch block is involved in the pathogenesis of secondary mitral regurgitation, caused by the dyssynchronous depolarisation of the ventricular walls. Karvounis et al. [55] observed in their study that after cardiac resynchronisation therapy, the severity of functional mitral regurgitation decreased acutely. This improvement was obtained due to the amelioration of the PMs’ nearby myocardial wall systolic deformation or the PMs’ own motion.

Dysfunction at any level of the mitral valve apparatus could impair the process of closing and opening the MV and could therefore generate mitral regurgitation. Papillary muscle dysfunction is frequently caused by acute or chronic myocardial ischemia and can lead to mitral regurgitation and eventually LV failure [12].

## 7. Mitral Valve Closure

### 7.1. Secondary Mitral Regurgitation

Papillary muscles are complex entities serving as part of the pathophysiology of various diseases. Taking into account the etiology of secondary mitral valve regurgitation, this includes coronary artery disease, dilated cardiomyopathy, and hypertrophic cardiomyopathy (HCM), which lead to PM displacement, asynchrony, mitral annulus dilation, impaired LV contractility and pathologic remodelling [56,57].

To emphasise the importance of PMs in mitral valve opening and closure, the study of Kanzaki et al. [58] highlights a problem concerning a reduction in the degree of MV regurgitation immediately after cardiac resynchronisation therapy. Often, patients with left bundle branch block and heart failure with reduced ejection fraction will benefit from cardiac resynchronisation therapy. In the mentioned study [58], the researchers performed a strain mapping of mechanical activation in the LV walls, assigning a corresponding part of the wall to each PM. The activation delay between the two PMs was 12 ± 8 ms in normal subjects, 106 ± 74 ms in patients before cardiac resynchronisation therapy and 35 ± 31 ms after resynchronisation therapy [58]. Also, it was observed that mitral regurgitation induced by PM dyssynchrony appeared earlier and was longer in duration before resynchronisation therapy. As a conclusion, the degree of mitral valve insufficiency was improved shortly after resynchronisation therapy. The reduction in activation time delay between the two PMs was the most important factor attributed to the improvement of mitral regurgitation. Also, the development of mitral insufficiency was correlated with the time delay of mechanical activation observed on strain mapping in the LV segments [58].

An experimental study coordinated by Levine [59] revealed that inducing ischemia in the inferior wall will cause mitral regurgitation due to restricted leaflets, but extending the ischemia to the PM which causes contractile dysfunction, the degree of mitral insufficiency will decrease despite the larger ischemic area. The induced ischemia of the PM lengthened the PM, improving the apposition of the leaflets, paradoxically decreasing the mitral regurgitation. This provides further proof that PMs are directly involved in the opening and closure of MV, as well as PM dysfunction and geometrical changes in the LV which can worsen or improve mitral valve insufficiency (Figure 4) [59].

### 7.2. Hypertrophic (Obstructive) Cardiomyopathy

Mitral valve prolapse and hypertrophic cardiomyopathy (HCM) are part of the arrhythmogenic syndromes with risk of sudden cardiac death [34,60]. Isolated PM hypertrophy is an uncommon variant of HCM [60,61,62]. Harrigan et colleagues [63] showed that almost 20% of patients with HCM and normal LV mass (with localised increased wall thickness) also presented an increased mass in the PMs. Likewise, a more substantial PM mass was associated with elevated LV mass index in this category of patients [63]. Although isolated PM hypertrophy is not diagnostic of HCM, it can be part of the phenotypic expression of the disease and it can contribute to dynamic LV obstruction, or it can, in association with other abnormalities, be involved in the pathogenesis of mitral regurgitation [64].

The most common cause of dynamic LV obstruction in HCM is represented by asymmetrical septal hypertrophy that leads to systolic anterior movement (SAM) of the mitral leaflets [65]. Mid-cavity obstruction as a result of solitary PM hypertrophy, direct PM insertion into anterior mitral leaflet and anterior displacement of PMs in the LV have also been reported as potential contributors to LV obstruction [62,63,65,66,67]. In addition, it was observed that beside the anterior displacement of the PM in HCM, the leaflet elongation of the mitral valve, especially the posterior one, can also contribute to the SAM phenomenon [68]. Under the circumstances, PM hypertrophy can induce a series of clinical features due to obstruction of LV mid-cavity, ranging from asymptomatic to shortness of breath, thoracic pain and even syncope or sudden cardiac arrest [65]. Also, PM hypertrophy and accessory PM proved to be significantly corelated with sudden cardiac death [67].

**Figure 5:**
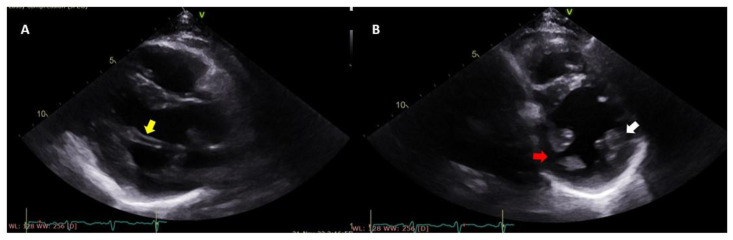
(**A**) PPM in parasternal long axis view (yellow arrow) in a patient with mitral valve prolapse. (**B**) Parasternal mid-ventricular short axis view at the level of PMs. Notice that PPM has two heads (red arrow), while APM has one (white arrow). (PPM: postero-medial papillary muscle; APM: antero-lateral papillary muscle, PM: papillary muscle).

**Figure 6:**
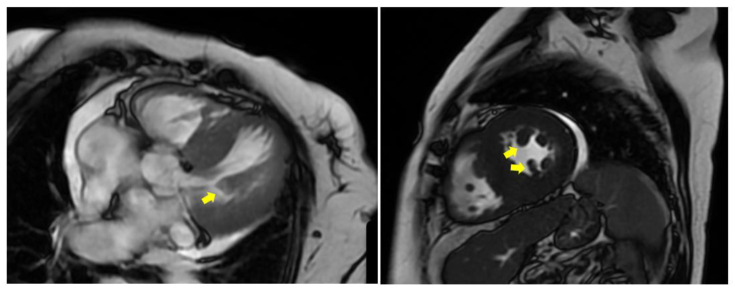
Cardiac MRI. PM hypertrophy in a patient with HOCM (yellow arrows). (MRI: magnetic resonance imaging, HOCM: hypertrophic obstructive cardiomyopathy, PM: papillary muscle).

For the evaluation of PM anomalies and morphology, ECG, echocardiography (Figure 5) and cardiac magnetic resonance (CMR) (Figure 6) are considered valuable diagnostic tools, with emphasis on the last one, that has proven to be superior to echocardiogram for their assessment [61]. Subsequently, CMR not only demonstrates that PM are a fragment of the cardiomyopathic process in HCM due to morphological characterisation, but is also able to identify and describe remodelling through fibrosis with the use of post-gadolinium T1 mapping [63,69]. Accordingly, Cresti et al. showed that CMR was able to identify fibrosis of the PM’s head and confirmed the presence of strictly localised cardiomyopathic process in HCM subjects [69].

Papillary muscle free strain is attracting growing interest in recent research [70,71,72]. It is known that PMs are frequently abnormal in HCM [73], and while CMR is not yet widely accessible, echocardiographic strain of the papillary muscle could be a valuable predictor of sudden cardiac death (SCD) in both low- and high-risk patients with HCM. This method is easily reproducible and can complement CMR as an additional prognostic tool [71].

The identification of fibrotic process at the level of PM has both diagnostic and prognostic value and is also a marker of proarrhythmic susceptibility. That given, the presence of a higher percentage of fibroblasts in the PM leads to changes in their membrane potential under cell-to-cell stretching, predisposing them to electrical anomalies as a response to altered mechanical conditions [74]. This was backed up by Gao and colleagues [75], who described the proarrhythmic effects of various pharmaceutical agents on human cardiomyocytes due to their interaction with neighboring fibroblasts. Additionally, it appears that the PMs of experimental animals with hypertension and heart failure exhibit a higher grade of fibrosis [76] as they are also predisposed to electrical disturbances. One explanation could be that increased cardiomyocyte-fibroblast electrical coupling in the initial stages of heart disease may impact the electrical activity of the cardiac human cells and precipitate arrhythmias [74,75].

Regarding ECG patterns amidst patients with isolated PM hypertrophy, there is a wide spectrum of abnormalities, ranging from normal ECG, prominent U wave, LV hypertrophy (the most frequent), negative/biphasic precordial T waves, and sinus arrhythmia to ST-segment alterations [65,67,77,78]. Correspondingly, Alsaud and colleagues [61] described a case of elongated and prominent anterolateral PM that generated electrocardiographic changes masquerading ST-segment elevation myocardial infarction. Allegedly, even if isolated PM hypertrophy may be overlooked by routine transthoracic echocardiography, occasionally an unjustified ECG pattern of LV hypertrophy can be explained by an isolated PM hypertrophy [67,69].

## 8. Conclusions

From a clinical perspective, the importance of the PM is mainly related to cardiac arrhythmias and secondary mitral regurgitation. Papillary muscle arrhythmias are probably under-recognised and arrhythmias with this origin are difficult to ablate. Establishing a successful ablation strategy depends on accurate noninvasive localisation of the arrhythmia source for preprocedural planning. Currently 12-lead ECG is the main instrument available for arrhythmia location. Regarding their role in MV closure, the PMs play an important role in secondary mitral regurgitation which has a ventricular mechanism in patients with both ischemic and nonischemic heart disease. Alterations in the geometry and function of the PMs as well as dyssynchrony play a role in PM-mediated secondary mitral regurgitation.
